# Protocol for a prospective multicenter longitudinal randomized controlled trial (CALIN) of sensory-tonic stimulation to foster parent child interactions and social cognition in very premature infants

**DOI:** 10.3389/fped.2022.913396

**Published:** 2023-01-16

**Authors:** Cassandre Guittard, Alexandre Novo, Julien Eutrope, Corinne Gower, Coralie Barbe, Nathalie Bednarek, Anne-Catherine Rolland, Stéphanie Caillies, Gauthier Loron

**Affiliations:** ^1^ Université Reims Champagne-Ardenne, C2S, Reims, France; ^2^ CHU Nantes, Département de Psychiatrie, Les Apsyades, Nantes, France; ^3^ Université de Reims Champagne-Ardenne, C2S, CHU Reims, Service de Pédopsychiatrie, Reims, France; ^4^ CHU Reims, Unité d’Aide Méthodologique, Reims, France; ^5^ Université de Reims Champagne-Ardenne, Research on Health University Department, C2S, Reims, France; ^6^ Université de Reims Champagne-Ardenne, CReSTIC, CHU Reims, Service de Médecine Néonatale et de Réanimation Pédiatrique, Reims, France

**Keywords:** neurodevelopment, preterm, brain, proprioception, interactions, clinical trial, brain imaging, parenting (MeSH)

## Abstract

**Introduction:**

Premature birth is associated with long-term somatic and neurological disorders, including cognitive, social and behavioral impairments. Moreover, the mothers of infants born preterm exhibit a higher prevalence of anxiety and depressive symptoms after birth. Early rehabilitation, developmental care, and parenting support have already been shown to have a positive impact on neurological outcome. However, no randomized controlled study has so far assessed the effects on parenting and long-term neurological outcomes of proprioceptive stimulation to trigger positive brain plasticity in very preterm babies. The CALIN project will therefore investigate the impact of sensory-tonic stimulation (STS) of extremely preterm infants by their parents on child parent interactions, infants' morphological and functional brain development and subsequent cognition (including social cognition), and parents' anxiety and depressive symptoms in the postpartum period.

**Methods and analysis:**

Infants born between 25 and 32 weeks of gestation will be randomly assigned to the “STS + Kangaroo care” or “Kangaroo care” group. The primary endpoint, child and parent interactions, will be rated at 12 months corrected age using the Coding Interactive Behavior system. Secondary endpoints include: 1/functional and anatomical brain maturation sequentially assessed during neonatal hospitalization using electroencephalogram (EEG), amplitude-integrated EEG (aEEG), cranial ultrasound and MRI performed at term-corrected age, 2/social and cognitive outcomes assessed at 15 months, 2, 4 and 6 years, and 3/parents' anxiety and depressive symptoms assessed at 7 ± 1 weeks after birth, using dedicated questionnaires.

**Ethics and dissemination:**

This study was approved by the French Ethics Committee for the Protection of Persons on 18 October 2021. It is registered with the French National Agency for the Safety of Medicines and Health Products (ANSM; no. 2020-A00382–37). The registry number on ClinicalTrials.gov is NCT04380051.

## 1. Introduction

### 1.1. Background

In 2018, the World Health Organization (WHO) estimated that 15 million children are born prematurely each year worldwide. The number of newborns surviving a premature birth has gradually increased over the years (1, 2). However, many of those will exhibit varying degree of neurological impairment. In the recent decades, the clinical and morphological picture of brain insults due to prematurity has changed. Classically, extensive clastic injuries (e.g., cystic necrosis of white matter, large infarction of brain parenchyma) most often led to cerebral palsy and/or moderate to severe cognitive impairment. Since the 1990s, however, their incidence has dwindled, and they have been supplanted by less prominent and more diffuse brain damage leading to the loss of vulnerable cells and impaired brain development (3–5).

Nowadays, the spectrum of neurodevelopmental outcomes of preterm babies broadly includes deficits in language, gross and fine motor skills, behavior, and cognition (especially executive functions and social cognition). The severity of the clinical picture is related to the degree of prematurity (6–12). Many brain areas undergo a sensitive period of development during the third trimester of gestation and after birth, during which sensory input shapes neurological maturation and future function. This is the case of the median prefrontal cortex, temporoparietal junction and posterior temporal sulcus (involved in cognition and social cognition) (13–15), the cerebellum, as well as visual, olfactory and somatosensory pathways (16). Premature birth dramatically modifies the context of this developmental window. During hospitalization, the developing brains of infants born preterm are exposed to stimuli that may be detrimental to their maturation (17) with either too much or too little sensorial input (18). This is referred to as *dystimulation*.

Furthermore, premature birth disturbs early infant-parent interactions, and ultimately relationships with others. Preterm infants are not only separated from their parents, but placed in a stressful, technical, and potentially painful environment. Comorbidities and sedation have a negative physiological impact and reduce their availability for interaction. On the parental side, the idealized postnatal period is replaced by an anxious–and even traumatic–experience (19–21). Parents often express guilt and anxiety about the survival of their child and their parenting skills. A higher prevalence of parental anxiety, postnatal depression and posttraumatic stress disorder has been observed in the mothers of infants born preterm (22), even up to 18 months after birth (23).

Finally, prematurity severely disturbs the ability of both parents and newborns to interact and find reassurance. More than 35% of children born preterm subsequently exhibit insecure attachment behavior in relationships with others (24). Given that studies have demonstrated a longitudinal link between attachment security and cognitive development (25, 26), specially social cognition (27, 28), it seems important to study the effects of disturbed early interactions on child development, and to test the potential of intervention programs to efficiently minimize these effects.

Developmental care has been implemented in many neonatal intensive care units (NICUs) to overcome the difficulties described above. The “Kangaroo mother care” and the “Newborn Individualized Developmental Care and Assessment Program” (NIDCAP) are among the most studied and practiced in the world. Fundamentally, those approaches place the family at the center of the newborn care, promoting affective contact with parents, and aiming at reducing dystimulation and sleep wake cycle interruptions (29). Those methods have shown positive effects on weight gain, sleep wake cycle interruptions electroencephalographic (EEG) activity, duration of hospitalization, some items of long term cognitive development and maternal anxio-depressive symptomatology (30–34). The involvement of parents in care is associated with many benefits: for the parents, it enhances the parenting process, their perception of their child, their sense of competence, and their attachment; for the child, it has a positive impact on cognitive and motor development, on executive function disorders and psychological disorders (31, 35–37).

Several teams are now investigating whether the focused enrichment of infants' sensorial input provides an additional effect to developmental care. Exposure to sounds such as maternal heartbeat, voices and music are promising avenues (38–42). Some studies have documented effect of somesthetic stimulation (43, 44). These interventions variably associate skin contact, massage (sliding on the skin), kinesthetic stimulation (movement of the child's limbs) and positioning. In most experimental protocols, those interventions have been performed one to three times a day, after feeding, during 5 to 10 days, on moderately preterm babies (39). Those interventions were associated with an increase in weight gain, lymphocyte natural killer activity (associated with a reduction of the incidence of late neonatal sepsis), as well as a decrease in pain response. A reduced length of stay in hospital and a better developmental score at 12 and 24 months were reported (43, 45–47). However, studies evaluating longitudinally the effectiveness of early tactile and kinesthetic interventions on several key aspects of extremely preterm children development are still sparse.

### 1.2. Objectives and hypotheses

The overall aim of this study is to assess effect of a sensori-tonic stimulation (STS) provided by parents, associated with kangaroo care, vs. kangaroo care alone, on the quality of parent-infant interactions, parental wellbeing, and ultimately brain maturation and cognitive outcome of infants born preterm.

#### 1.2.1. Primary objective

This study will investigate the benefits of early STS, provided by one of the parents on its infant born preterm on the developing interactions between them. We expect to observe stronger interactions in the dyads who practiced STS + kangaroo care, than in the control group (kangaroo care only).

#### 1.2.2. Secondary objectives

The present work will assess, in children born very preterm, the effect of “STS + kangaroo care” vs. “kangaroo care” alone on: 1/the morphological and functional brain maturation, 2/the precursors of cognitive development at 15 months, 3/psychomotor development at 2 and 4 years, and 4/cognition including social cognition assessed at 6 years of age.

Moreover, this study will assess the impact of the intervention on symptoms of anxiety and depression experienced by the parents of children born preterm.

## 2. Methods

### 2.1. Design

The CALIN study is a prospective, multicentric randomized study. The clinicians who carry out the assessments will be blind to the result of the randomization. Participants will be recruited in French tertiary neonatal centers equipped with Neonatal Intensive Care Units.

### 2.2. Participants

#### 2.2.1. Study population

Children born between very preterm and their parents will be eligible if they meet the following inclusion criteria: 1/the child is inborn, 2/birth between 25 and 32 weeks of gestation and weight over 600 g at birth, 3/the child's hospitalization at the recruiting center is planned until 36 weeks corrected age, 4/the child's parents have parental authority and have agreed to participate in the study by signing the informed consent, 5/the child's parents are available physically and mentally to participate in the study and 6/the child and his/her parents are affiliated to the social security system.

The exclusion criteria are the following: 1/the child is hemodynamically unstable, 2/the child has a suspected comorbidity (e.g., genetic syndrome, congenital malformation, brain injury, skin pathology), 3/the child was born from a multiple pregnancy, 4/the child was born anonymously, 5/the child is to be separated from his/her parents (e.g., assigned to infancy protection services).

If the child's condition does not allow the practice of STS (for example: hemodynamic compromise, enterocolitis), the intervention may be temporarily discontinued until the condition improves. The discontinuation and the resumption of the intervention are decided by the medical staff in charge of the child, who informs and explains the decision to the parents. Newborns will not be excluded from the intervention group.

#### 2.2.2. Sample size calculation

The sample size has been calculated so that a two-tailed test will detect a significant difference in the primary endpoint (Coding infant behavior scale at 12 months corrected age, see below), with a power of 90% and a significance level of 0.05 (alpha value). Forty-eight infants would be required in each group (NQuery 4.0® software). In anticipation of loss to follow-up, the target size has been increased by 20%, such that 60 children will be included in each group, resulting in inclusion of a total of 120 infants.

#### 2.2.3. Randomization

The newborns and their parents will be randomly assigned to either the experimental group or the control group. Those in the experimental group will participate in the STS intervention, in addition to kangaroo care. The control group will benefit from kangaroo care alone. Besides that, the medical and nursing care will be the same, regardless of the outcome of the randomization.

### 2.3. Intervention

Sensory-tonic stimulation (STS) refers to a protocolized, tactile, vestibular and kinesthetic stimulation, provided by a parent to her/his baby born preterm. This proprioceptive stimulation is carried out using an enveloping and continuous touch with a moderate pressure, as described in the literature (48, 49).

STS will be adapted to the newborn's term and level of development, broadly stratified into three stages (25-30, 31–34 and 35–36 corrected age, respectively). The preterm infant is either installed in a microbead baby nest in the incubator or on the changing table, depending on the newborn's usual arrangement and maturity. This position facilitates the sensory-tonic care, the observation of the behavior of the newborn, her/his signs of comfort and discomfort, and her/his state of alertness. At each stage, the stimulation begins with a two-handed enveloping contact with the child lying on his or her back: one hand on the trunk, the other hand supporting the pelvis, with the legs folded. Then, the hands slowly slide under the child's head/neck and pelvis, to lift the child and perform vestibular stimulation with gentle rocking motions. As the child matures, the parent adds hand movements along the child's limbs, then slow flexion-extension movements. The approximate duration of each step is between fifteen seconds and one minute, depending on the baby's tolerance and the step considered. Moving on to each next step requires a favorable state of alertness and the absence of signs of discomfort. As much as possible, the contact and gentle pressure between the hands and the baby's skin are continuously maintained. At all times, if signs of discomfort are observed: a comfortable and calming position is maintained (usually lateral roll-up).

Parents will be coached by a trained, designated professional, initially using a mannequin. They will also be shown a training video and given an illustrated training booklet covering all the steps of STS. They will begin from the 10th day after birth and when the parents feel ready to perform it and will continue until discharge from hospital or 36 weeks corrected age. STS should be carried out 5 times per week, 5-15 min each time. STS will be performed when the newborn exhibits quiet alertness (State 3 or 4 according to Brazelton (50)). The intervention will be interrupted if signs of discomfort are detected in the newborn or his/her parents. Once a week, a trained psychologist or psychomotor therapist will supervise the parent's gestures and discuss his/her experience.

“Skin to skin care” will be practiced without any restriction in both groups, following local policies. In the experimental group, newborns and their parents will participate in STS in addition to kangaroo care. The time, number, duration of each skin-to-skin care or STS will be collected, as well as somatic stability, initial and maximal state of alertness of the baby (51).

Preterm babies will benefit from the usual neonatal intensive care, following the local policies of each center.

### 2.4. Assessments and outcomes

**Figure 1:**
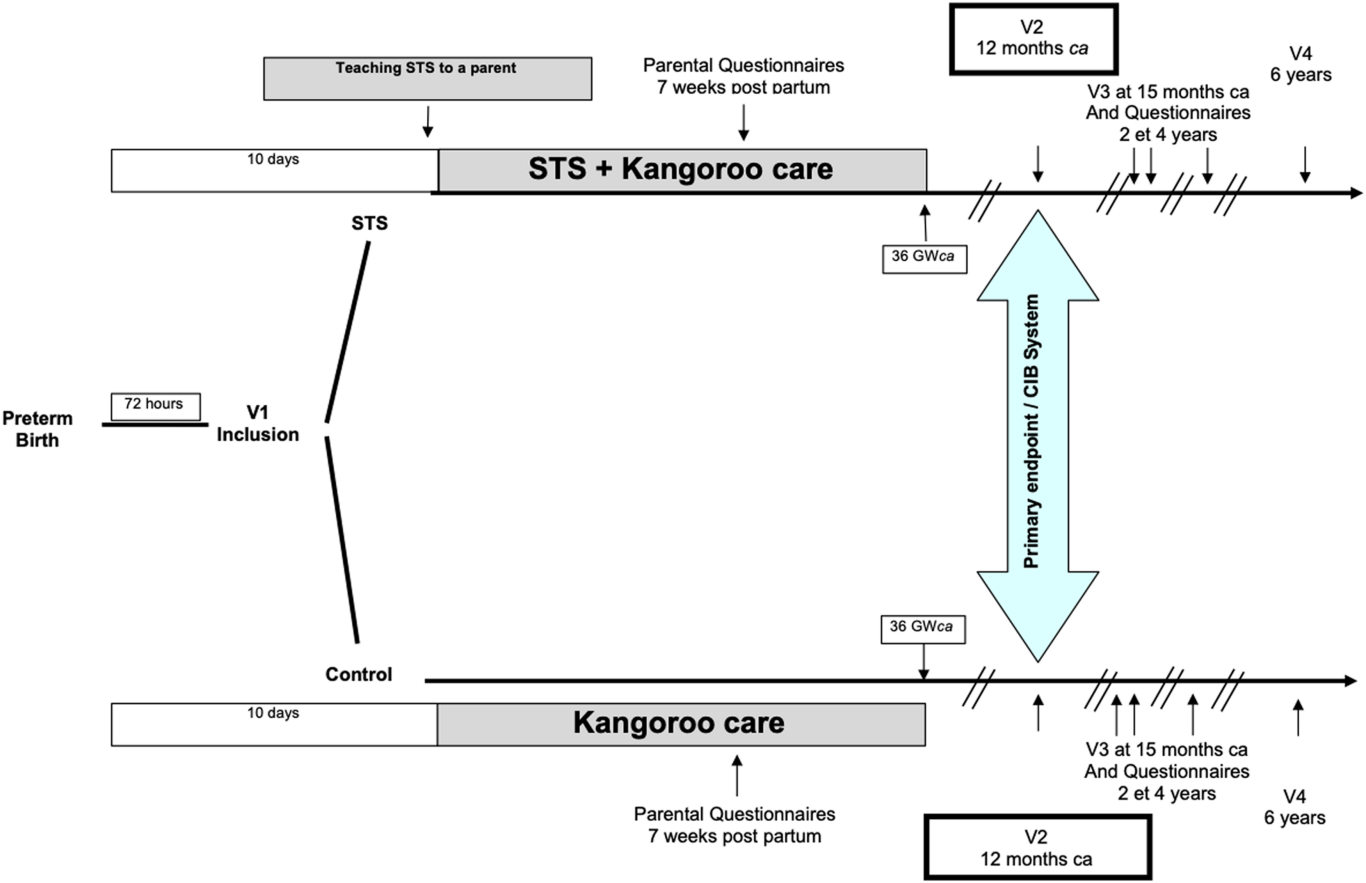
Design of the study, overview of assessments and outcomes.

An overview of the different assessments and outcomes is provided in Figure 1.

#### 2.4.1. Primary outcome

The quality of interactions between the very premature infants and their parents will be scored at 12 months corrected age using the Coding Infant Behavior system (CIB) (52). The latter assesses the quality of child-parent interactions by observing the occurrence of behaviors along six dimensions: parental sensitivity, parental intrusion, child's social engagement, child's negative emotionality and engagement, dyadic reciprocity, and negative dyad states. This scale is validated from birth to 3 years of age and has shown a great sensitivity in scoring interactions in many social and/or pathological situations (53). Early CIB scores have previously been correlated with cognitive development (54). We will record two 15 min sequences of child-parent interactions during free play. Observations will be double-quoted by professionals trained to use the CIB, blinded to the randomization group. The main judgment criteria will be the score in “child's social engagement” dimension of the CIB system. The Secondary judgment criteria will be the scores in the other dimensions of the CIB system.

#### 2.4.2. Secondary outcomes

##### 2.4.2.1. Morphological and functional development of the brain

All the data yielded by the following paraclinical examinations will be anonymized and sent to the main investigating center for centralized review. They will be interpreted by two experts for each technique used, blinded to the randomization group.

Serial cerebral ultrasound scans will be performed during the infants' NICU stay, following a standardized protocol. Lesions will be classified: 1/according to the Papille classification for intraventricular hemorrhages, 2/according to the De Vries classification for periventricular leukomalacia (55). Moreover, several two-dimensional measurements will be performed on ultrasound scan to assess brain growth: interhemispheric distance, ventricular width, thickness of motor and somatosensory cortex, diameter of thalami and cerebellum.

Brain magnetic resonance imaging (MRI) will be performed at term-corrected age including a diffusion sequence, following a standardized protocol of acquisition. Imaging will be interpreted according to Kidokoro (56). Moreover, regional brain volumes will be quantified using semi-automatic, post-hoc segmentation.

EEG and amplitude-integrated EEG will be sequentially recorded. Timing of the EEG recordings and their interpretation will follow the guidelines of the French society of electrophysiology (57).

##### 2.4.2.2. Sequential assessment of cognition and psychomotor development

Precursors to cognitive development will be assessed at 15 months corrected age using the “Batterie d’Évaluation Cognitive et Socio-émotionnelle” (58). This psychometric tool assesses cognitive and socio-emotional development in young children up to 24 months, based on standardized activities and standardized sequences of interactions between the psychologist and the child.

Child development at 2 and 4 years will be measured using the Ages and Stages Questionnaire (ASQ 24 and ASQ 48) (59). Those questionnaires, filled by parents, include 30 items assessing five areas of development: communication, gross motor skills, fine motor skills, problem solving, and individual or social skills. A French, validated translation is available, featuring age-appropriate questions corresponding to the child's expected level of development and normative data.

Cognitive and social development will be assessed at 6 years using an extensive set of neuropsychological tests and theory of mind tasks: 1/Global intellectual abilities will be assessed with the Wechsler Intelligence Scale for Children (WISC-V). This scale has five main indices: Verbal Comprehension, Visual Spatial, Fluid Reasoning, Working Memory, and Processing Speed and allows calculation of a global intellectual quotient. 2/To assess attention, inhibition and mental flexibility, The NEPSY's Auditory Attention and Associated Responses subtest will performed (60). 3/The NEPSY's Sentence Repetition subtest will be used to assesses phonological working memory abilities through a task where participants have to repeat sentences of increasing length and complexity (60). 4/The Test of Everyday Attention for Children (TEA-CH)'s Opposite Worlds subtest will attention and inhibition abilities through a pointing task in response to a verbal cue (61). 5/The Raven Matrix Test assesses nonverbal intellectual efficiency through a reasoning by analogy task (62). 6/Social cognition will be assessed using Cognitive and Affective theory of mind abilities tasks (63–69).

##### 2.4.2.3. Parental anxiety and depressive symptoms

Anxiety and depressive symptoms will be assessed in both parents at 7 weeks ± 1 after birth. Maternal anxiety and depressive symptoms will be measured using the State Trait Anxiety Inventory (STAI-Y), Parental Stress Index (PSI), Beck Depression Inventory (BDI), Edinburgh Postnatal Depression Scale, and Modified Perinatal Post Traumatic Stress Disorder Questionnaire. As some of these scales are not suitable for rating fathers, they will be replaced with non-gender specific scales. Paternal anxiety and depressive symptoms will therefore be measured using the STAI-Y, PSI, BDI, and Posttraumatic Stress Disorder Checklist for DSM-5 (70–75).

### 2.5. Data analysis

First, we will carry out a descriptive analysis of the data, with means and standard deviations, and counts and percentage for the quantitative and qualitative variables, respectively. For all subsequent analysis, the group (experimental vs. control) will be the independent variable. The scores obtained on each scale will be the dependent variable.

#### 2.5.1. Statistical analysis of primary outcome

The main judgment criteria (score in the “child's social engagement” dimension of the CIB at 12 months) will be compared between the experimental group and the control group using a Student or Mann-Whitney test, depending on the application conditions.

Secondary judgment criteria (scores in the other dimensions of the CIB at 12 months) will be compared between the experimental group and the control group using a Student or Mann-Whitney test, depending on the application conditions.

#### 2.5.2. Statistical analysis of secondary outcomes

All secondary outcomes are quantitative measures. For all these measures, we will compare the scores of the experimental group with those of the control group, according to the distribution of the data, with parametric or nonparametric tests (Student's *t*, Mann-Whitney, chi^2^ or Fisher's exact tests), depending on the application conditions. Correlations will be calculated between quantitative measures.

A *p* value of < 0.05 will be considered statistically significant.

Statistical analysis will be performed with software “R®”(76).

## 3. Discussion

Although prematurity has been identified as a *public health problem* by the WHO since 2010, studies assessing the efficiency of early intervention programs are still sparse.

Developmental care and the kangaroo method (also called *skin-to-skin care*) have been implemented in many neonatal intensive care units (NICUs). These programs aim to reduce dystimulation, sleep-wake cycle interruptions and enhance affective contact with parents. They have been shown to have many positive effects on weight gain, somatic development, electroencephalographic (EEG) activity (77), medical complications, and duration of hospitalization (30, 78, 79).

Several teams are now investigating whether the focused enrichment of infants' sensory environment can improve sensory input and, ultimately, brain development. Massage and exposure to sounds such as maternal heartbeat, voices and music are promising avenues that are currently being explored (39, 40, 80, 81).

The present study will focus on sensory-tonic stimulation (STS). Complementing the standard care designed to protect premature newborns from all the usual dystimulation, the purpose of this STS intervention will be to improve proprioceptive input, which is generally lacking in NICUs. The sensorimotor cortex exhibits activity-dependent plasticity during this crucial period of development (82, 83). In the past, tactile stimulation of very premature infants in the form of *massage* has had a positive impacts on physical growth (e.g., weight, height) (48, 84), electroencephalographic (EEG) activity maturation [35,37], sleep quality and stress behaviors (e.g., crying, motor agitation) (43, 48), physiological measurements (e.g., IGF binding protein-3, glucose, insulin, cortisol and thyroid hormones) (84), and early psychomotor development (85, 86). Interestingly, this kind of intervention may also have a positive impact on parents’ moods and skills. Tactile, tender and affectionate contact increases plasma levels of oxytocin and endorphins (associated with feelings of wellbeing and happiness) in both the infants and their parents (43). However, these studies are based on different protocols, sample sizes, participants (parents vs. caregivers), and stimulation duration.

One of the strengths of the CALIN study is to consider different dimensions of the child's development within its family, analyzing the parents' mood, early interactions, psychomotor and long-term cognitive development, and brain maturation. These different dimensions seem to interact with each other over time.

We are aware that the CALIN protocol has potential limitations and biases. We may be challenged by potential variability in stimulation time if parents continue to practice STS after the infant has left hospital. We also expect to a risk of loss to follow-up due to the longitudinal design of this study.

## Data Availability

Publicly available datasets were analyzed in this study. This data can be found here: ClinicalTrials.gov NCT04380051.
