# Corrigendum: Protocol for a prospective multicenter longitudinal randomized controlled trial (CALIN) of sensory-tonic stimulation to foster parent child interactions and social cognition in very premature infants

**DOI:** 10.3389/fped.2024.1386605

**Published:** 2024-02-26

**Authors:** Cassandre Guittard, Alexandre Novo, Julien Eutrope, Corinne Gower, Coralie Barbe, Nathalie Bednarek, Anne-Catherine Rolland, Stéphanie Caillies, Gauthier Loron

**Affiliations:** ^1^ Université Reims Champagne-Ardenne, C2S, Reims, France; ^2^ CHU Nantes, Département de Psychiatrie, Les Apsyades, Nantes, France; ^3^ Université de Reims Champagne-Ardenne, C2S, CHU Reims, Service de Pédopsychiatrie, Reims, France; ^4^ CHU Reims, Unité D’Aide Méthodologique, Reims, France; ^5^ Université de Reims Champagne-Ardenne, Research on Health University Department, C2S, Reims, France; ^6^ Université de Reims Champagne-Ardenne, CReSTIC, CHU Reims, Service de Médecine Néonatale et de Réanimation Pédiatrique, Reims, France

**Keywords:** neurodevelopment, preterm, brain, proprioception, interactions, clinical trial, brain imaging, parenting (MeSH)

A Corrigendum on Protocol for a prospective multicenter longitudinal randomized controlled trial (CALIN) of sensory-tonic stimulation to foster parent child interactions and social cognition in very premature infants By Guittard C, Novo A, Eutrope J, Gower C, Barbe C, Bednarek N, Rolland A-C, Caillies S and Loron G (2023). Front. Pediatr. 10:913396. doi: 10.3389/fped.2022.913396

In the published article, an author's name was incorrectly written as Cailles. The correct spelling is Caillies.

The authors apologize for this error and state that this does not change the scientific conclusions of the article in any way. The original article has been updated.

